# Structural Racism, Geographies of Opportunity, and Maternal Health Inequities: A Dynamic Conceptual Framework

**DOI:** 10.1007/s40615-025-02345-5

**Published:** 2025-03-03

**Authors:** Irene Headen

**Affiliations:** Department of Community Health and Prevention, Drexel University Dornsife School of Public Health, Philadelphia, PA 19104 USA

**Keywords:** Maternal health, Structural racism, Racial inequities, Neighborhood environment, Systems thinking, Conceptualization

## Abstract

Addressing the grave racial inequities in maternal health requires a new generation of research that better operationalizes root causes of these outcomes. Recent frameworks improving the conceptualization of structural racism have illuminated the need for better conceptual clarity when investigating neighborhoods as a site of structural marginalization for Black birthing populations as well. In particular, better conceptualization of dynamic feedback in how neighborhoods are constructed and experienced, especially as they embed vicious cycles of place-based racialization, is integral to producing conceptually relevant and translatable evidence to address inequities in Black maternal health. This study presents a newly developed framework that integrates dynamic insight on neighborhood contexts from multiple disciplines to better conceptualize how it operates during the childbearing window to drive inequitable maternal morbidity rates among Black birthing people. I also compare and contrast this framework with existing frameworks based on how they represent key domains of social and structural determinants, neighborhood context, and dynamic feedback. Illustrating the strengths and weaknesses of each framework can improve researchers’ ability to leverage these frameworks when developing project-specific conceptual models on structural racism, neighborhood context, and Black maternal health. Building a comparative repository of frameworks, in conjunction with developing new frameworks, will improve the field’s capacity to follow best practices of rooting research in conceptually explicit models that improve operationalization and translation of evidence to eventually eliminate racial inequities in maternal health.

## Introduction

Worsening rates of maternal morbidity and mortality in the US place these outcomes at the top of the national public health agenda [1, 2]. Black birthing people^1^ have persistently higher rates of maternal mortality (MM) and severe maternal morbidity (SMM), both in comparison to national rates [3, 4] and compared to their white counterparts [5–7]. The most recent estimates place MM as 2.6 times higher [4] and SMM as approximately 1.9 times higher [8] for Black birthing people compared to their white counterparts. While MM is more severe than SMM—which denotes a near-miss, critical, but non-fatal outcome—approximately 20–30 SMM cases occur for every one maternal mortality case [9]. The severity of increasing rates and widening inequities across both of these outcomes has sparked a wealth of research to identify points of intervention across clinical, environmental, interpersonal, and social contexts [1, 10, 11]. However, the translation of existing evidence into action has not achieved desired goals, as reflected in increases in overall rates of these outcomes documented in the Healthy People 2030 objectives [12–14]. As a result, calls within the field have emphasized the need for renewed research efforts that identify novel intervention targets, better contextualizes the structural constraints facing communities, and more consistently centers equity [2, 12].

In conjunction with these calls, there has been increasing attention to the need for clear conceptual frameworks and models^2^ to guide emerging work in this area [12, 15]. In particular, health equity scholars have emphasized the importance of distinguishing between social determinants of health, which are defined as the conditions in which people are born, grow, live, work, and age [11, 16], and structural determinants of health, which consist of the upstream norms, practices, institutions, and policies that define how social determinants are distributed for different socially positioned groups [17–19]. Distinguishing between these distinct but interconnected processes is especially important as they relate to racial inequities in maternal and infant health outcomes [15, 20, 21] to better understand structural racism as a root cause [19]. Because it operates as a multi-dimensional, dynamic process [22, 23], clear conceptual frameworks and models are critical for operationalizing interactions within and between specific domains and understanding how they give rise to processes of racialization at particular time frames over the life course. Explicitly measuring place-based structural racism—through domains such as residential segregation, inequitable divestment in essential services or economic opportunity, and inequitable distributions of greenspace and recreational areas resulting from zoning practices to give a few examples—allows us to capture distinct processes of embodiment that operate in conjunction with the social determinants of health [17, 18]. Additionally, because these structural processes are explicitly designed to reinforce racial hierarchies [23, 24], they are embedded within Black and Brown neighborhoods independent of the opportunity structure of that neighborhood [22, 25, 26].

Robust conceptual models are needed to guide work investigating neighborhood context as one of the upstream domains of structural racism contributing to racial inequities in maternal morbidity and mortality [27–29]. In particular, research in this area using social determinants to proxy structural exclusion has produced mixed findings that have been attributed to a range of factors (e.g. geographic location, selection of study population, geographic level of observation, time period, and neighborhood measures used) [17, 28, 30]. However, few studies present a clear conceptual model guiding their hypotheses for how neighborhood factors interact with each other in general and how they operate specifically to impact inequities in adverse maternal outcomes. Neighborhoods exist and are embodied as a system emerging from the confluence of factors across built, social, and environmental domains [26, 30, 31]. Thus, the same individual factors may have a different impact on health depending on how they operate in relation to the other elements within the neighborhood environment. This has implications for both evidence generation as well as translation into action. Depending on the characteristics of the neighborhood system, intervening on one domain or factor may be more or less effective depending on the reinforcing or constraining relationship with other neighborhood factors, especially for those that facilitate processes of racialization [23, 26, 30]. As such, clear conceptual models can support a more effective translation of evidence into action [30, 32].

In this paper, I briefly review the neighborhood and maternal morbidity and mortality literature, discuss insights from the field of systems thinking and system dynamics that better conceptualize neighborhood environments from a dynamic perspective especially concerning processes of racialization, briefly summarize existing conceptual models and frameworks in relation to how they incorporate dynamic perspectives, and present a conceptual framework developed to incorporate the systems literature and address gaps in understanding the dynamic interplay between neighborhood characteristics and mechanisms that drive racial inequities in maternal morbidity in the US. This novel conceptual framework distinguishes between the social and structural domains of neighborhood context, clearly conceptualizes reinforcing feedback processes that create vicious cycles of racialized exclusion from opportunity and accumulation of disadvantage, and connects these processes to the critical window of pregnancy for maternal health. I conclude by emphasizing important takeaways to aid in use of this conceptual framework in research going forward.

## Neighborhood Context and Maternal Morbidity and Mortality

A well-established body of literature links neighborhood environment to adverse perinatal outcomes [33–36]. This literature indicates consistent associations between higher neighborhood adversity (e.g. socioeconomic deprivation, low food access, low walkability, lack of housing affordability, pollution, physical disorder, and crime) and increased risk of both preterm birth and low birth weight [34, 36, 37]. Neighborhood environment and outcomes for the birthing parent have been less widely researched [38], but indicate that neighborhood adversity, assessed using a variety of domains as indicated above, increases the risk of adverse gestational weight gain patterns [39], hypertensive disorders during pregnancy [35], gestational diabetes mellitus [40], maternal depression [41], and postpartum weight retention [42]. Recent work investigating neighborhood environment and either SMM or MM, however, suggests mixed findings. Studies report positive associations between neighborhood socioeconomic deprivation, as a specific domain within neighborhood adversity, and increased risk of SMM, although a subset of studies indicate an inverse relationship [11, 43, 44]. Other studies have used multiple indicators of neighborhood context beyond socioeconomic deprivation; associations are not consistent across indicators with some studies reporting no association between neighborhood context and SMM [38].

Across these studies, neighborhood context has been operationalized in numerous ways. Most studies within this body of literature focus on social determinants clustered within neighborhood environments based on the assumption that this clustering creates distinctly inequitable geographies of opportunity [29, 30, 45]. For example, many studies solely rely on individual or composite measures of socioeconomic context such as income, employment, household structure, and reliance on public assistance [11, 35, 38]. Some studies expand the domains of the social determinants represented to include measures of housing, crime, and access to health care resources as well [43, 46]. This approach reflects two key assumptions. First, that socioeconomic context is a complete or mostly complete proxy for the cumulative lack of access to neighborhood resources and exposure to neighborhood stress that characterize the main mechanisms through which adverse health outcomes emerge [17, 29, 30]. Second, this approach implicitly assumes that measuring the summed impact of neighborhood adversity across social determinants represents the *structural* processes that differentially distribute these resources by racialized group [24, 26, 47]. However, this assumption is challenged by studies that report associations between neighborhood social determinants and SMM are more attenuated among Black populations [39, 42–44], which is also reflected in literature on other perinatal outcomes as well [34, 48]. Neighborhoods that Black populations live in may be more similar to each other in terms of measures of structural racism than white neighborhoods, even if the opportunity structures of these neighborhoods differ based on the social determinants of health.

The burgeoning body of work focused on structural indicators of neighborhood context in relation to SMM has largely focused on measures of racial residential segregation [49–53]. Across an array of segregation indicators—including racial composition, race-based isolation index, redlining, and the index of concentration at the extremes based on race, income, and racialized income—about half of studies find associations with SMM, especially among Black birthing people. Other indicators of structural racism, such as incarceration inequality, education inequality, and employment inequality, report less consistent associations, with most of these indicators suggesting no significant association with SMM [28, 46, 54]. These measures have set the foundation for investigating structural racism and maternal health, but there is general acknowledgment in the field that they do not capture the full theoretical complexity of structural racism, which can limit the actionability of research using only measures of racial residential segregation. Calls to better align measures with the theoretical foundations of structural racism, including multidimensionality, interconnected and reinforcing relationships, and historical grounding [17, 24], can be facilitated first by improving the conceptual models that are leveraged to guide the operationalization of structural racism in maternal health research.

## Neighborhoods as Dynamic Systems

As stated above, one key part of refining conceptual models representing structural indicators of neighborhood context is better incorporating the interconnections between neighborhood characteristics that synergistically create contexts of marginalization. We can look to systems-based approaches to better illuminate these dynamic interconnections in ways that emphasize processes of racialization impacting neighborhood typologies [22, 32]. Key examples from disciplines adjacent to public health emphasize the importance of incorporating dynamism into understanding neighborhood context [55–57], especially in relation to systems of racialized oppression in urban environments. Dynamism in the systems context refers to the patterns of interrelationships or interactions between factors contributing to a particular problem over time that are reciprocal in nature and create emergent behaviors that change how to understand and act on that problem over time [58]. At their core, the three examples presented in this section that represent dynamism present neighborhoods as flows of people and investment driven by neighborhood characteristics which make them more or less attractive for different “actors” or residents within an urban space [59, 60]. For example, young professionals may flow into densely populated neighborhoods with many recreational amenities and rental properties as they navigate their early career life stage, which in turn attracts the flow of those types of investments (e.g., restaurants and cafes) to those neighborhoods [57]. However, the neighborhood resource profile that mid-career and family-oriented populations prioritize may include owner-occupied housing and spacious environments with greenspace. Ideally, they would identify neighborhoods to align with this shift in desire or find ways to align their existing neighborhoods with their changing needs [57]. Influencing these general patterns of people and resource flows, processes of racialization intertwine racialized stereotypes of resident populations with processes of investment and resource allocation which, in turn, feedback to impact the “attractiveness” and resources access, and thus flows of investment and people into a neighborhood.

Sewell [56] demonstrates the necessity of incorporating a racialized lens in her analysis of racial residential segregation as a dynamic, spatialized process resulting in differential exposure to constrained choice and resources for racially marginalized populations. In particular, the accumulation of institutional racism across the systems within neighborhoods produce concentrations of racially marginalized populations into various types of “risk-scapes” rather than healthful environments. Risk-scapes consist of factors such as credit deprivation (both home and business), poor and declining housing quality, low social capital, and lack of access to health promoting resources, whereas healthful environments are more likely to include factors such as robust organizational services, supportive built environments, and concentrations of social and economic capital [56]. Thus, it is not necessarily due to the physical segregation of populations itself, but the accumulating impacts of racialization and isolation that happen through co-location of these processes in place. As an example, Sewell (2015) emphasizes how the real estate system produces a reverberating impact of racism across institutional systems which in turn constrain the presence and deployment of neighborhood assets. She describes a feedback system that relies on the magnitude of applicant-based credit deprivation being contingent on the racial or economic composition of the neighborhood. Subsequently, neighborhood differences in mortgage credit deprivation propagate differences in resources across other service domains based on assumptions of “credit worthiness” at the neighborhood level. This, in turn, drives cycles of divestment that are amplified by homeowners not being able to invest in improving the value of their homes. An emergent property of these institutionalized mechanisms within the field of real estate is a process of isolation for racialized neighborhoods that amplifies their exclusion from opportunity over time in a self-perpetuating way. Stated more explicitly, residential segregation must occur in conjunction with processes of racism in order to confer negative health effects [56]. Absent those racist processes, residential segregation can emerge from mechanisms that, while race-based to a degree (e.g., spatial assimilation as Sewell states; [52, p.95]), alternatively, have positive impacts on health, as reflected in literature on ethnic and immigrant enclaves and health [61–64].

The Living Cities National Community Development Initiative presents an applied example of how dynamism drives flows of people and investment across neighborhood contexts [57]. They create taxonomies of neighborhood “types” across four US cities using neighborhood characteristics similar to those used in public health: housing, job access, select types of consumption amenities (e.g. supermarkets), transit, and socioeconomic conditions. However, they conceptualize these factors as interdependent contributors to the emergent property of “neighborhood attractiveness.” Essential to neighborhood attractiveness is that it is more than the sum of the individual neighborhood “parts” and it is embedded in a feedback relationship with the population of residents in the neighborhood and the resources available over time. It can either drive positive cycles of attracting residents who in turn advocate for more resources which in turn increases the attractiveness of the neighborhood in an ongoing virtuous cycle, as demonstrated by the Living Cities Initiative’s “Coming Attraction” neighborhood type [57]. Alternatively, lack of neighborhood attractiveness can lead to transient resident populations who do not or are not able to advocate for positive resource accumulation resulting in a vicious cycle of decreasing neighborhood attractiveness over time, as demonstrated by their “Transit Underdeveloped” neighborhood type. As part of characterizing the interconnected relationship between neighborhood attractiveness and population flow, the authors also identify distinct “stage of life” communities that offer a set of amenities that appeal to certain demographics at certain stages of their life (e.g., childbearing and family rearing; [57]). There are two important implications from this Initiative’s demonstration of dynamic neighborhood types. First, neighborhoods are embodied differentially by populations residing in them based on the composition of resources, attractiveness, *and* propensity to change. Second, since neighborhood change is intertwined with neighborhood composition, the same intervention will have different impacts in different neighborhoods. Incorporating these dynamic insights is integral to better understanding and translating evidence on neighborhoods and health into action, especially for key stages of life.

Northridge and colleagues [55] add to understanding dynamism in the context of urban environments and explicitly link it to the production of health disparities. Their framework is designed to focus on the contributions of social determinants to the accumulation of environmental hazards driving disparities across multiple outcomes [55]. This framework explicitly illustrates the dynamic interplay between fundamental, intermediate, and proximate factors driving wellbeing and health outcomes. The authors focus on dynamism to elucidate mechanisms through which spatial concentrations of poverty and wealth lead to concentration of resources that influence policy, which in turn leads to the development of desirable built environment features such as easy access to services, accumulation of transportation, and siting of affordable housing. Concentration of desirable resources then feedback to attract those with higher income and wealth, further amplifying the concentration of political power and desirable resources. Furthermore, the authors emphasize how racism operates as a distinct fundamental cause driving the differences in the distribution of resources as well as the built and social environment factors to produce and maintain inequities across health outcomes. They conclude by emphasizing the necessity of including dynamism in conceptual frameworks in order to better understand the processes of self-reproduction, unintended consequences (i.e., increasing rather than decreasing inequities), and the importance of time delays in understanding how intermediate (and even proximate) factors feedback to impact fundamental causes, thus keeping the whole inequity producing system in place and resistant to disruption [55].

## Comparative Analysis Across Existing Conceptual Models and Frameworks

While the examples above demonstrate the importance of more clearly including dynamism in conceptualizations of neighborhood context and racial health inequities, existing conceptual models or frameworks vary in whether and to what extent they achieve this goal. In this section, I present a narrative comparison of conceptual models and frameworks that represent the domains of neighborhood context, social and structural determinants of health inequities, and dynamic feedback between neighborhood indicators. Literature was identified using the following search terms, “conceptual framework,” “conceptual model,” “structural racism,” “neighborhood,” “maternal health,” and “maternal morbidity,” through Google Scholar and PubMed. I also reviewed reference lists of identified and included articles. All selected articles were in English, included maternal or infant health outcomes, specifically focused on presenting a conceptual model or framework, and included a visualized diagram of their framework. The goal of comparison across identified conceptual frameworks and models was to illuminate how the models vary in representing each of the domains mentioned above and assess strengths and weaknesses in representation across all three domains of the constructs needed to guide research on racialized neighborhood context and maternal health outcomes.

Table 1 presents the key comparative elements across domains and their alignment with selected conceptual frameworks. For the social and structural determinants domain, I evaluated models and frameworks on whether they included only social determinants, social determinants as a proxy for structural processes of marginalization, social and structural determinants as distinct constructs, or both social and structural determinants with in depth descriptions of “multilevel, multidimensional” mechanisms at work [17, 19, 23]. For neighborhood context, I evaluated models and frameworks on whether they included it as a specific domain or not; if they did, I assessed whether it was discussed as aggregation of residents’ demographics, as single domain attribute(s) distinct from demographics, or as a set of multidimensional interconnected attributes creating neighborhood environment [29, 32, 45]. Finally, for representation of dynamic feedback, I evaluated models and frameworks on whether or not they included it at all. If they did, I assessed whether it was represented as only bi-directional feedback between pairs of variables; as multifactor (i.e., more than two variables) circular causal chains accompanied by general descriptions of nonlinearity, interconnectivity, reinforcing processes, or co-dependent relationships; or as multifactor circular causal chains accompanied by descriptions of specific mechanisms and/or feedback processes [31, 65].

**Table 1:** Comparison of conceptual frameworks across domains of social and structural determinants, neighborhood context, and dynamic feedback

**Legend (domains: classification categories):** Representation of social and structural determinants: • only social determinants; • social determinants as a proxy for structural determinants; • social and structural determinants included; • multilevel, multidimensional structural mechanisms described in depth		Representation of neighborhood context: • not present; • aggregation of demographic characteristics; • Inclusive of attributes beyond demographics, still relying on a single separate dimensions of neighborhood context (e.g., neighborhood disadvantage index) • attributes as separate from demographics included and discussed in multidimensional, interconnected mechanisms		Representation of dynamic feedback: • not present (i.e., linear relationships); • bidirectional relationships between pairs of variables; • multifactor (i.e., bidirectional or longer) circular causal chains, with general justification for use of nonlinear, or feedback approach; • multifactor, circular causal chains with specific descriptions of systems mechanisms and/or feedback processes	
Author and year	Summary of framework domains^1^	Representation of social and structural determinants	Representation of neighborhood context	Representation of dynamic or feedback relationships	Outcome
Headen	Foundational context: life course exposure Macrolevel: structural racism, structural marginalization across domains of -Race -Class -History -Migration Mesolevel: Neighborhood geography of opportunity across domains of availability: -Consumption -Resources -Built environment -Access to public -Services and accessibility: -Transportation -Social environment Individual opportunity across domains of: -Income -Education -Employment -Family -Socioeconomic status -Wealth -Marital status Individual-level: age, resource access, health behaviors, stress, stress response, access to Quality healthcare, prepregnancy health status access to quality perinatal healthcare, pregnancy complications	Description of multilevel, multidimensional structural mechanisms	Multidimensional interconnected attributes	Multifactor, circular causal chains with specific description of systems mechanisms and feedback processes	Maternal morbidity
Crear-Perry et al. (2021)	Macrolevel: Slavery, Jim Crow, 13th Amendment, Redlining, GI Bill Mesolevel: food stability, safety, rates of incarceration, access to care, housing, neighborhood demographics, income, education	Description of multilevel, multidimensional structural mechanisms	Aggregation of demographic characteristics	Bidirectional relationships between pairs of variables	Infant and maternal mortality
Chambers et al. (2021)	Macrolevel: structural racism characterized by the following elements: -Negative societal views, -Housing, -Hidden resources, -Law enforcement, -Medical care, -Employment, -Education, -Policing Black families, -Community infrastructure	Description of multilevel, multidimensional structural mechanisms	Multiple single-dimension attributes beyond demographics embedded within an aggregate neighborhood structure	Multifactor, circular causal chains with specific description of systems mechanisms and feedback processes	None specified although conducted among preconception, pregnant, and postpartum women
Carmichael et al. (2022)	Foundational context: reproductive health, health and racial equity, community-engaged research Macrolevel: historical context, structural/societal factors; Mesolevel: social determinants; Individual-level: life-course experience, health-related factors, clinical precursors	Description of multilevel, multidimensional structural mechanisms	Multiple single dimension attribute (i.e., neighborhood conditions) as part of a composite measure of social determinants	Not present	Severe maternal morbidity and inequities in this outcome
Simoncic et al. (2022)	Structural determinants and social determinants of health inequities Socioeconomic context and political context: governance, macroeconomic policies, social policies, public policies, cultural and societal values, neighborhood level deprivation, neighborhood level education, rural/urban; Individual characteristics: social status, ethnicity, marital status, partner education, partner employment, social cohesion & social capital, family socio-economic environment Socioeconomic position and social class: education level, occupation, income; Intermediary determinants and social determinants of health Biobehavioral and psychophysiological outcomes: material circumstances, behaviors lifestyle and biological factors, psychosocial factors and environment, psychological disorder, physiological disorder; health system	Description of multilevel, multidimensional structural mechanisms, relating specifically to those driving health inequities as well as overall health	Multidimensional, interconnected attributes	Multifactor, circular causal chains with specific description of systems mechanisms and feedback processes	Adverse birth outcomes mostly for newborn (maternal health included as it relates to infant health, e.g. Excessive GWG b/c of links to adverse infant outcomes)
Sonderlund et al. (2022)	Foundational context: structure of racial enactment through society, exposures to - mass media, - public health & health care, - housing, - criminal justice system, - education, - economic system, - environment/ climate change Macrolevel: segregation, police, conviction, sentencing, income inequality, spatial concentrations of incarceration Mesolevel: social network disruption, community stigma, crime, police, community breakdown, chronic stress proliferation, threat & victimization, community divestment; Individual level: allostatic load/weathering, health-risk behavior	Description of multilevel, multidimensional structural mechanisms	Multidimensional, but not explicitly interconnected in description	As multifactor circular causal chains, describing nonlinear, feedback processes	Adverse birth outcomes (including low birthweight, preterm birth)
Hailu et al. (2022)	Foundational context: life-course exposure Macrolevel: structural racism, institutional practices, bias/prejudice Mesolevel: scocioeconomic assets, psychosocial stressors & resources, physical environment Individual-level: health related behaviors, physiological wear & tear, clinical conditions	Description of multilevel, multidimensional structural mechanisms	Multidimensional, interconnected attributes	As bidirectional relationships between groups of variables	Maternal morbidity and mortality

^1^Key domains represented within conceptual frameworks are listed to provide context for our evaluation approach and organized by level of influence. Foundational context refers to the overarching framework, theory, or process that influences the way that factors at all additional levels operate. After foundational context, domains are organized by macrolevel factors, mesolevel factors, and individual level factors similar to the social ecological model. Not all frameworks have all levels

Frameworks focused on a broad range of perinatal health outcomes. Some focused on specific outcomes such as severe maternal morbidity [28, 66] or infant and maternal mortality [20, 28] while others broadly focused on adverse birth outcomes [37, 67]. All frameworks included social and structural determinants of health as well as multilevel, multidimensional descriptions of structural mechanisms, but varied in the level of detail included to characterize determinants. Some frameworks remained high level, focusing on general historical and contemporary racialized norms [66], while others named specific mechanisms of governance [37], policies [20], and institutional processes such as those mediated through mass media and criminal justice systems [67]. Three frameworks were developed with the goal of more clearly conceptualizing structural racism as a driving determinant of health inequities [20, 28, 68] and thus illustrated more detailed mechanisms compared to other frameworks. For example, Chambers et al. [68] obtained perspectives of structural racism from Black preconception, pregnant, and postpartum individuals and identified novel domains of structural racism through this research. They described domains of “hidden resources” and “policing Black families” that had either not been included or had be poorly conceptualized from the experiences of Black birthing people in previous frameworks [68].

All frameworks included neighborhood as a construct, with about half conceptualizing it as either aggregations of residents’ demographic characteristics [20] or aggregations of characteristics beyond demographics [66, 68], such as access to physical activity space or green space for community gardening [68]. The remaining frameworks [28, 37, 67] defined neighborhood context through multidimensional characteristics, with either implicit or explicit interconnections, resulting in an overarching, emergent neighborhood quality. Simonsic et al. [37], for example, build on the widely used WHO Social Determinants framework and operationalize neighborhood as the interplay between sociodemographic factors, education level, rural/urban status, and other environmental amenities, such as, “pleasant views, good schools, or green space” ([37], p 20), that create an overall “neighborhood attractiveness.” All of the frameworks that operationalized neighborhoods as multidimensional and interconnected attributes [28, 37, 67] explicitly linked spatialized distribution of neighborhood characteristics to macrolevel processes of structural marginalization.

All but one framework [66] included dynamism or feedback in some form to define relationships between factors included. Two frameworks represented feedback as only bidirectional relationships between pairs of variables [20, 28] and three represented feedback as multifactor, circular causal chains [37, 67, 68]. Including feedback as multifactor, circular chains between factors is important because it better reflects the complexity of social and structural mechanisms driving health, which is relevant for better representing complex processes of racialized marginalization. Among three frameworks that included multifactor, circular causal chains, two additionally anchored descriptions in specific systems mechanisms and feedback processes [37, 68] were included. For example, Chambers et al. [68] emphasize that Black birthing people reported negative societal norms which impacted access to resources and housing, in turn impacting employment and educational access. This then impacted access to healthy environments and healthcare resources, which all fed back into the negative societal norms and views. This vicious cycle characterized the structural racism that participants felt trapped within [68].

Overall, these frameworks illustrate an array of approaches to represent social and structural determinants, neighborhood context, and dynamic feedback when investigating racial inequities in adverse maternal and infant health outcomes. A main focus of most frameworks identified was to more clearly distinguish between the social and structural determinants at the root of inequities, so they were most consistent in distinguishing between social and structural determinants and emphasizing the multilevel and multidimensional nature of these factors. Frameworks were less consistent in how they represented neighborhood context and dynamic feedback, demonstrating an array of mechanistic pathways illustrated to capture these constructs (Table 1). Looking across all three domains, only one framework [37] included enough conceptual detail in each to potentially bridge the remaining theoretical gaps discussed in “Neighborhood Context and Maternal Morbidity and Mortality,” especially regarding the need to include dynamism in conceptualizations of how structural processes generate maternal health inequities. However, even this framework largely focused on detailing dynamic feedback processes in downstream domains, interconnecting the social determinants of health with the psychological, physiological, and behavioral pathways that ultimately result in adverse birth outcomes. Thus, the need to more comprehensively incorporate dynamic feedback throughout the mechanistic processes driving maternal health inequities, especially within upstream domains such as neighborhood context, remains.

## Conceptual Framework Incorporating Dynamic Feedback into Neighborhood Systems

**Fig. 1:**
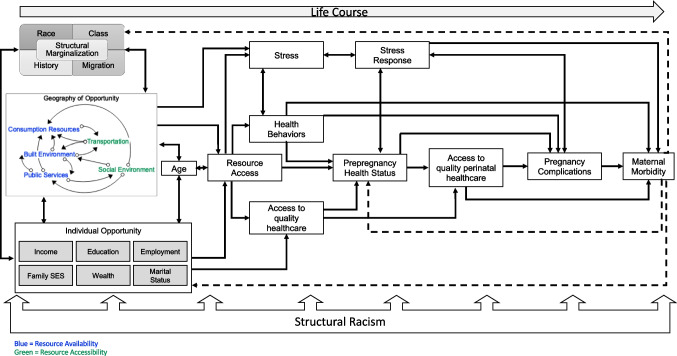
Conceptual framework of structural marginalization, dynamic neighborhood systems, and racialized maternal morbidity outcomes

Leveraging the dynamic insights described above and addressing the gaps in existing frameworks, I developed a conceptual framework to integrate multi-dimensional neighborhood context, spatial processes of racialization, and racial inequities in maternal morbidity (Fig. 1). This framework builds on previous conceptual models and frameworks [31, 69, 70] but specifically elaborates on the dynamic feedback mechanisms relevant for conceptualizing neighborhood context, bringing in insights discussed in “Neighborhoods as Dynamic Systems” and “Comparative Analysis Across Existing Conceptual Models and Frameworks.” It aims to illustrate how these pathways are dynamically shaped by processes of structural racialization, which refers to the way in which social hierarchies by racial categories are used to prescribe meaning and access to opportunity which in turn reinforce the ascribed tropes to members of that racial group [22], creating a vicious cycle of structured marginalization leading to adverse health outcomes.

Anchoring the framework are multilevel contributors to access to opportunity mediated through individual and neighborhood contexts. These multilevel contributors, which are described in detail below, include factors such as individual income and education, access to healthcare, neighborhood context and resource accessibility, and upstream domains of structural marginalization. Starting with neighborhood context, as previously described, there is a well-established relationship between attributes of the neighborhood environment, working both independently and jointly, to impact adverse pregnancy outcomes [11, 38]. In this framework, neighborhood environment is defined by the presence and dynamic interaction of resources across the consumption/service domain (including things such as access to retail, services, schools, museums, and dining), the built environment (including access to green space, pollution, housing quality, and food environment), and the access to public services (such as access to school, recreational facilities, and healthcare) as well as the structures that facilitate access to those resources, most centrally transportation and factors in the social environment (including social cohesion, civic participation, crime and safety, and socioeconomic composition) [45, 57, 69]. The framework distinguishes between availability, the presence or absence of a resource or attribute, and accessibility, the ability of residents to access an attribute, for two reasons [25, 45]. First, it is important to emphasize the need for both elements to be present in order to deploy the benefits, or experience the harm, of neighborhood attributes. The co-location of these two elements, availability and accessibility, varies across different racialized contexts as Sewell [56] denotes above. Second, the dynamic feedback between these two elements can structure the accumulation of civic and political power over time, which Northridge et al. [55] previously discussed. The concentration of political power can either ensure that residents can self-actualize the spatial environments that support their needs or, alternatively, create downward spirals of resource-poor, divested neighborhoods. Neighborhood quality, thus becomes an emergent property of the “geography of (in)opportunity” reflected in the accumulation of particular feedback relationships that interlink attributes across domains of availability and access in the neighborhood environment.

Next, these geographies of (in)opportunity in turn attract (or confine) residents based on their individual sociodemographic characteristics as represented in the “individual opportunity” domain [37, 38, 69]. The influence of these social determinants on residents’ sorting into neighborhoods has been well established [32, 59, 71]. In particular, they reflect the ways in which having a favorable profile across these factors allows individuals and families to better align desired goals for neighborhood quality and amenities with actualized outcomes. However, having an unfavorable profile can stymie individuals’ and families’ ability to actualize the desired context in which they wish to live. The accumulation of individual factors across the domains of income, education, employment, family SES, wealth, and marital status at the residential population level in turn also strongly impact the concentration of political power [55] and social capital [56] that facilitate the deployment of neighborhood resources as well as the acquisition of new or modification of existing resources to support the residential populations’ desired opportunity structure.

The co-constructed individual and neighborhood access to opportunity is constrained by processes of structural marginalization, which is defined here as a more generalized process of societally structured exclusion from resources and opportunity based on hierarchies of social identity at the macrolevel [22]. These hierarchies can be defined across many different social identities and processes, and this framework focuses on domains of race, class, history, and migration because of how they intersect to create specific spatialized patterns of opportunity (e.g., ethnic enclaves and redlining) [24, 56]. The combined impact of social constraint structured by these domains is conferred through norms, policies, and practices that in effect “discount” the potential gains that can be obtained from specific geographies of opportunity and individual opportunity profiles, both separately and together [19, 45, 56]. For example, neighborhood environments with similar geographies of opportunity can have different levels of actualized resource access or deployment, and subsequently health impacts, if they are predominantly Black and impacted by structural racism compared to predominantly immigrant communities impacted by segregation processes that have a more positive impact on social connections or other factors, as described above [26, 56].

The upstream, multilevel social and structural determinants of opportunity access in this framework mediate resource access operating over the life course to generate a particular deployment of those resources to impact health leading into the childbearing window. Age, as indicated in the life course model that anchors this framework [72], is also an integral part of understanding patterns of accumulation of social determinants of health (included in the individual opportunity domain) and their deployment to shape resources access. Age is included to represent its role in how individuals and families seek out geographies of opportunity aligned with their particular life stage, as discussed in “Neighborhoods as Dynamic Systems” in the exploration of “stage of life” neighborhood typologies from the Living Cities project [57]. Age can be understood as a chronological, social, and biophysiological process (described below), all of which is integral to how racialized neighborhood contexts are transduced into adverse maternal health outcomes [73]. Along this line, the conceptual framework reflects a process of embodiment widely established as a key mechanism in the process of transduction of health outcomes [32, 70]. Specifically, resource access impacts stress, stress response, and health behaviors to drive prepregnancy health status through biobehavioral and psychophysiological mechanisms such as stress activation through the hypothalamic–pituitary–adrenal (HPA) axis, social and behavioral coping approaches, allostatic load, and weathering leading to advanced biological aging [21, 70, 74]. Access to quality healthcare also mediates the ways in which resource access is transduced into health status prior to pregnancy [2, 12, 75]. Once becoming pregnant, prepregnancy health status, stress/stress response, and access to quality care over the perinatal period cumulatively define the risk of pregnancy complications [76]. Pregnancy complications, in conjunction with stress and access to quality healthcare over pregnancy and the perinatal period, result in the occurrence of maternal morbidity.

The pervasive impact of structural racism across the childbearing process characterizes the disproportionate risk that Black people and people of color experience across the cascade of events (and racialized contexts) leading to maternal morbidity [15, 77, 78]. For example, long-standing implicit and explicit bias as well as institutional racism in the healthcare system impacts the quality of Black birthing people’s care [2, 78, 79]. Black birthing people routinely report experiencing discrimination in healthcare contexts that accumulates to structurally constrain their access to treatment and impacts approaches to medical intervention, such as increased rates of cesarean section among Black patients [79, 80]. That in turn increases both the risk of complications and, subsequently, resource needs in ways that compound existing differential access to resources. Additionally, access to and type of health insurance, mediated through inequitable employment opportunities and/or experiences of poverty for Black communities, can further limit access to providers and quality care locations [10, 53]. Contributing to these institutional limitations and structural barriers, legacies of place-based segregation continue to limit the spatial access to maternal and perinatal healthcare in predominantly Black neighborhoods [78, 81].

The final part of this conceptual framework returns to the importance of dynamic feedback throughout these processes. Maternal morbidity feeds back to impact prepregnancy health status of a birthing person, should they experience a subsequent pregnancy, and their subsequent need for and deployment of individual socioeconomic capital to support recovery [76]. In particular, resources that might have been directed towards other needs, such as housing, food, or social needs in response to the expansion of the family, must be redirected to support medical costs associated with recovery from a maternal morbidity event as well as additional social support and resource needs to navigate the adjustment to the postpartum period when birthing person and baby are experiencing significant health needs [82]. There is also feedback represented in the framework between maternal morbidity into the continued racialization of Black birthing people and other people of color by reinforcing persistent stereotypes anchored in historical racism [77, 78]. For example, the stereotype that Black individuals’ reproduction needs to be controlled through either surveillance, intervention (e.g., coercive contraceptive approaches), or victim-blaming is rooted in legacies of enslavement and Jim Crow and may be reified in response to continued maternal morbidity events for Black birthing people in ways that differ from other racial/ethnic groups [15, 77, 78].

## Discussion

I present a conceptual framework to inform the investigation of neighborhood context and racial inequities in maternal health outcomes that improves the conceptualization of dynamic feedback as a key part of mechanisms driving this relationship. Most importantly, I pulled from systems-based literature on neighborhood dynamics to better represent 1) social *and* structural determinants of adverse maternal health outcomes, 2) specific mechanisms underlying neighborhood as a domain within the ecosystem of structural racism, and 3) feedback relationships that characterize how factors are interrelated to produce an infrastructure of racial exclusion for Black birthing people. A few important takeaways emphasize key advances that the framework contributes and important considerations for adoption and use of this framework to guide research to understand neighborhood context and maternal morbidity inequities, which are discussed below.

The first takeaway is that composite measures of neighborhood factors will better operationalize neighborhood context. Interdependence between neighborhood factors mean that trying to operationalize them separately will obscure synergistic effects that emerge from the co-location of factors within neighborhood context. This may be especially important for understanding maternal health outcomes in racially marginalized populations given the interaction with structural marginalization that further constrains or discounts potential benefits of positive neighborhood factors [26, 30]. Various composite measures of neighborhood environment across domains of influence have emerged in recent years, as reflected by the use of vulnerability indices [83] and opportunity indices based on geography of opportunity frameworks [25, 84, 85]. However, scholars have reiterated the importance of anchoring development of these composite measures in both clear theoretical foundations, and community experience in order to better understand their impact [84, 85]. In particular, indices have been shown to inconsistently classify the same neighborhoods, and not acknowledging the conceptual foundations driving development of a measure may lead to unintended or unexpected findings when translating evidence into action [85], p759).

A second takeaway for applying this conceptual framework is that measuring processes of structural marginalization in a spatialized way is needed to understand inequities in health outcomes. In particular, macrolevel processes of structural marginalization across race and history can modify the meaning and potential benefit of particular social determinants that co-occur within a neighborhood geography. Furthermore, specific geographies of opportunity can influence processes of structural marginalization depending on their spatial organization. An example of this is reflected in the ways that current patterns of racial residential segregation have feedback relationships with land-use policies that in turn impact access to resources [26]. As such, understanding spatial organization of geographies of (in)opportunity provides further insight into how processes of structural marginalization create distinct inequitable landscapes and health outcomes for populations by race [45, 85].

A third takeaway is that methods that enhance the ability to represent feedback processes are key to understanding the role of neighborhoods in maternal health inequities. Systems-based approaches have been increasingly called upon to better understand health inequities and neighborhood contexts [31, 86]. In particular, there are a wide array of systems tools that can bridge from conceptual frameworks, such as the one presented here, to both quantitative and qualitative analyses as well as operate across the continuum from evidence generation to implementation [87, 88]. For example, Brittin et al. [89] use system dynamics, which combines qualitative system mapping with quantitative computer simulation [86], to improve insight around urban interventions to reduce chronic disease inequities. Leveraging published studies, they develop a system map of neighborhood context and chronic disease inequities, emphasizing the interconnected feedback between neighborhood factors such as housing capacity, social cohesion, and neighborhood attractiveness. Based on this system map, they integrate data from the Census with estimates from published studies to create a simulation model that tests key policy interventions representing real world neighborhood interventions to improve chronic disease inequities. Their findings indicate that because of the feedback between neighborhood factors, joint interventions on income/employment and social cohesion/neighborhood attractiveness are needed to see meaningful reductions in chronic disease inequities; interventions on either domain separately are not successful [89]. In another example, Lemeke et al. [90] use a community-based system dynamics approach to understand the impact of structural racism on maternal healthcare, engaging with key stakeholders throughout the process. Focusing on access to care and healthcare quality, they developed a qualitative system map of factors driving structural racism and impacting maternal mortality in Black women [90]. In contrast to the quantitative simulation approach that Brittin et al. [89] implemented, Lemke et al. [90] build qualitative insight around policy interventions, asking stakeholders what interventions they perceived to be most important given the system map of Black maternal mortality they had created. Participants again indicated that joint interventions, rather than single or sequential interventions, across factors ranging from doula care to addressing bias within the healthcare system were needed to achieve the desired reduction in inequities [90]. These examples illustrate how combining systems approaches with existing public health and epidemiologic approaches can lead to more effective and inclusive insight into intervention approaches when dynamic conceptual frameworks are used [31].

Despite these potential benefits of applying systems based conceptual frameworks, such as this one, to better conceptualize feedback processes, neighborhood context, and structural racism, there remain challenges to consider when adopting for use in research. In particular, the time scale over which neighborhood environments change may be longer than the duration of a pregnancy. As Brittin et al. [89] present in their work, neighborhood change and chronic disease outcomes develop over similarly extended time frames such that embodiment of exposure to adversity in the neighborhood context accumulates in ways that drive incremental development of long-term poor health outcomes. However, for an outcome with a shorter time scale, such as pregnancy, the physical attributes of a neighborhood, or resource availability, might not change on the same time scale for any one individual pregnancy. As such, this conceptual framework is better suited to contextualize population-level processes and research questions rather than parse research delving into individual level etiology. Furthermore, this issue of time scale means that domains driving resource accessibility, such as transportation and the social environment, may be more prominent in distinguishing how neighborhood becomes embodied to produce adverse pregnancy outcomes. This becomes a particularly relevant given that pregnancy itself is a dynamic balance of resource need and resource access, which then becomes exacerbated if complications arise [82]. As such, research questions well suited for inquiry informed by this conceptual model include (1) formative questions about what domains and what relationships between domains need to be included in development of future composite measures of place-based structural racism; (2) etiologic questions focusing on ways in which neighborhood and health care access jointly shape maternal morbidity inequities; and (3) contextual questions investigating how resource access may be modified by different racialized neighborhood contexts to deferentially impact maternal morbidity outcomes.

Addressing the challenge of time scales is also important for understanding how this framework can be used to translate evidence into action. If intervention targets, for example, are developed to address transportation barriers to health care access, they may have unanticipated impacts on access to other necessary consumption resources (such as food) or the built or social environment that may, over time, actually *reduce*, the ability of the neighborhood’s geography of opportunity to support healthy pregnancy outcome. For example, if promoting paid or discounted ride-share services [91] to overcome transportation challenges in getting to health care appointments over time reduces overall ridership of public transportation that would go to main food retail locations, this may have an impact on how staffed that transportation line is or whether it remains in use, thus having the unintended consequence of *reducing* accessibility of neighborhood resources rather than increasing it. As such, implementation-oriented research questions relating to what types of neighborhood-level interventions and how particular interventions may operate in real-world contexts are well suited to understanding with this conceptual framework, as long as explicit articulation of anticipated time scales is incorporated to ensure effective contextualization of evidence and action.

## Conclusion

Conceptual frameworks are key to ensuring theoretical consistency when navigating the etiology to action continuum in research and improving operational clarity of studies [92, 93]. In particular, they can aid investigators in developing project-specific conceptual models that represent the hypothesized and testable mechanisms being pursued in a specific project [93]. This is critical to overcoming the “conceptual inconsistency” [32, 50] that has led to heterogenous findings and been cited as a barrier to continued progress in generating research on structural racism to eliminate racial inequities in maternal health. My conceptual framework is specifically developed to improve the incorporation of dynamic or feedback mechanisms in the investigation of neighborhood context and structural racism on racial inequities in maternal morbidity outcomes, which has been a barrier to developing theoretically informed measures of place-based structural racism in this area of research. Applying the framework to existing heterogenous findings going forward can support better understanding of the sources of heterogeneity across studies on neighborhood factors and maternal health inequities. Additionally, it can facilitate more intentional measurement, modeling, and estimation of such measures going forward. Because of its more detailed representation of dynamic feedback, my framework can also be used to explore unanticipated consequences when intervention in one domain of neighborhood contexts propagates change across other interconnected domains [31]. This complements an ongoing push within the implementation science field to use comprehensive frameworks that acknowledge the complex relationships between factors beyond the research context to improve implementation of interventions, programs, and policies at different scales [55, 94], which is particularly relevant to making much needed headway on equitably improving outcomes such as maternal morbidity.

## Data Availability

Not applicable.

## Footnotes

1 The term “birthing people” is used as a gender-inclusive reference to individuals who are capable of becoming pregnant and giving birth. This includes women, non-binary individuals, trans-men, and other gender expansive individuals. This paper interchanges between the use of “birthing people” when referring to the population of interest and “maternal” when referring to the outcomes in order to remain consistent with how outcomes are identified throughout the literature.

2 Paradies and Stevens (2005) offer a general definition of conceptual diagrams as a diagram of proposed relationships among a set of concepts, factors or variables about a particular hypothesis, question, context, problem or topic.” (p. 1012) Brady et al. (2020) further refine distinctions between conceptual models and conceptual frameworks as conceptual models being visualizations that encapsulate what a research team has prioritized and chosen to study and is intentionally focused in scope. In contrast, conceptual frameworks encapsulate what is possible to study and is intentionally comprehensive.
